# Amount, Distribution, and Quality of Protein Intake Are Not Associated with Muscle Mass, Strength, and Power in Healthy Older Adults without Functional Limitations—An *enable* Study

**DOI:** 10.3390/nu9121358

**Published:** 2017-12-14

**Authors:** Anne Gingrich, Alexandra Spiegel, Robert Kob, Daniel Schoene, Thomas Skurk, Hans Hauner, Cornel C. Sieber, Dorothee Volkert, Eva Kiesswetter

**Affiliations:** 1 Institute for Biomedicine of Aging, Friedrich-Alexander-Universität Erlangen-Nürnberg, Kobergerstraße 60, 90408 Nürnberg, Germany; Alexandra.Toelke@fau.de (A.S.); robert.kob@fau.de (R.K.); daniel.schoene@fau.de (D.S.); cornel.sieber@fau.de (C.C.S); dorothee.volkert@fau.de (D.V.); eva.kiesswetter@fau.de (E.K.); 2 Chair of Nutritional Medicine, Technical University of Munich, Gregor-Mendel-Str., 85354 Freising—Weihenstephan, Germany; skurk@tum.de (T.S.); hans.hauner@tum.de (H.H.); 3 Institute of Nutritional Medicine, Klinikum rechts der Isar, Technical University of Munich, Georg-Brauchle-Ring 62, 80992 Munich, Germany; 4 Krankenhaus Barmherzige Brüder Regensburg, Prüfeninger Straße 86, 93049 Regensburg, Germany

**Keywords:** protein intake, protein distribution, aging, muscle strength, muscle power

## Abstract

To maintain muscle mass in older age, several aspects regarding the amount and distribution of protein intake have been suggested. Our objective was to investigate single and combined associations of daily protein intake, evenness of protein distribution across the three main meals, number of meals providing ≥0.4 g protein/kg body weight (BW), and number of meals providing ≥2.5 g leucine, with muscle mass, strength, and power in successful agers. In this cross-sectional study in 97 healthy community-dwelling adults without functional limitations aged 75–85 years, protein intake was assessed using 7-day food records. Muscle mass, leg muscle strength, leg muscle power, and handgrip strength were measured according to standardized protocols. Mean daily protein intake was 0.97 ± 0.28 g/kg BW and the coefficient of variance between main meals was 0.53 ± 0.19. Per day, 0.72 ± 0.50 meals providing ≥0.4 g protein/kg BW and 1.11 ± 0.76 meals providing ≥2.5 g leucine were consumed. No correlations between single or combined aspects of protein intake and skeletal muscle index, leg muscle power, leg muscle strength, or handgrip strength were observed (Spearman’s *r* of −0.280 to 0.291). In this sample of healthy older adults without functional limitations, aspects of protein intake were not associated with muscle mass, strength, or power.

## 1. Introduction

Aging is accompanied by a progressive loss of muscle mass, muscle strength, and muscle power, leading to functional decline [1,2]. The maintenance of muscle function is crucial in terms of enabling mobility and independent living. Compared with young adults, in older individuals, muscle protein synthetic response to anabolic stimuli, i.e., exercise and the ingestion of protein, particularly to small amounts of protein, is attenuated [3,4]. To overcome this ‘anabolic resistance’ [5] and to preserve muscle mass as well as strength, several aspects of protein intake are discussed as being relevant: daily amount, distribution over the day, per-meal amount and protein quality [6,7,8,9,10]. 

Large longitudinal cohort studies in older adults have shown that dietary protein intake is positively associated with lean body mass, physical function, and muscle strength [11,12,13,14]. Therefore, a daily protein intake of at least 1.0 g/kg body weight (BW) per day has been suggested for healthy older adults to maintain lean body mass and function [7]. 

Addressing the distribution of protein intake over the day, a randomized 7-day crossover feeding study in younger adults showed 25% higher muscle protein synthesis (MPS) rates when dietary protein was evenly distributed compared with distribution in a skewed pattern [15]. While these results were not observed in two experimental studies in older adults [16,17], two recent epidemiological studies reported a smaller decline in muscle strength and lean mass in older persons with a more evenly distributed protein intake [18,19]. Previous results of our group showed a more even protein intake pattern in robust older adults compared with that in frail older adults [20].

A saturable dose–response relationship between the amount of protein per meal and MPS rate has been observed in healthy older men: At a protein amount of 0.4 g/kg BW per meal, a plateau is reached and MPS is maximally stimulated, even to a similar extent as in young adults [3]. In recent analyses of National Health and Nutrition Examination Survey (NHANES) data, the number of meals per day with at least 30 g protein was associated with leg lean mass and muscle strength [21]. In contrast to the 30 g per meal approach, the per-meal threshold relative to BW is in line with the relative protein intake recommendations per day. Correspondingly, a per-meal concept of three daily meals providing at least 0.4 g protein/kg BW/meal was developed in order to maintain muscle mass [9]. To the authors’ best knowledge, there has been no study investigating whether adhering to this concept affects muscle mass or functional parameters in older individuals. 

In addition to the amount, the protein quality affects muscle synthetic response [22]. The quality of protein is characterized by the amino acid composition as well as the digestion and absorption kinetics [22]. Experimental studies with leucine-enriched meals showed that leucine has the potential to overcome anabolic resistance [23,24]. Hence, the leucine content of a meal is considered as a main determinant of its quality with regard to the stimulation of muscle protein synthesis. For healthy older adults, a per-meal amount of at least 2.5 g leucine has been suggested [7]. So far, no epidemiological studies have been published considering the amount of leucine per meal in relation to muscle mass, strength, or function. 

Previous epidemiological studies investigated only one of the four above-mentioned aspects of protein intake. Moreover, these studies focused on rather young-old populations with a mean age <70 years [12,14,18,21] and/or heterogeneous samples in terms of functionality and health status [12,14,20,21]. However, the risk of mobility disability and loss of independence is increasing with age [25,26]. Thus, healthy and, at the same time, functionally unimpaired community-dwelling persons aged 75 years or older are an interesting group in which to study lifestyle-related factors of successful aging. To our knowledge, the potential single and combined associations between these four aspects of protein intake and muscle mass as well as strength have not been investigated in a homogenous and carefully phenotyped sample of ‘successful agers’. Therefore, the present study aimed at investigating whether the protein intake pattern consisting of

1. daily amount of protein,
2. evenness of protein distribution across the three main meals,
3. number of daily meals providing at least 0.4 g protein/kg BW, and
4. number of daily meals providing at least 2.5 g leucine

is associated with muscle mass, muscle strength, and power in healthy community-dwelling adults aged 75–85 years without functional limitations. Based on previous findings, we hypothesized that these aspects of protein intake are positively associated with muscle mass, muscle strength, and power. 

## 2. Materials and Methods

### 2.1. Study Design and Participants

For this cross-sectional study, community-dwelling healthy Caucasian adults aged 75–85 years were recruited. The recruitment took place between April 2016 and April 2017 using the citizen registry of the city of Nuremberg, Germany. We aimed at recruiting 100 persons with equal proportions of men and women. Exclusion criteria were: BMI less than 18.5 or greater than 35 kg/m^2^, smoking, immobility, need of care, unintended weight loss of more than 5% in the previous three months, cognitive impairment (Mini Mental State Examination (MMSE) < 24 points), blood transfusion in the previous three months, and current participation in intervention studies. In addition, the following physician-diagnosed chronic diseases (self-reported, medication list) led to exclusion: human immunodeficiency virus infection, liver disease, diabetes mellitus, endocrine disease, autoimmune disease, renal failure requiring dialysis, lung disease, stomach ulcer, or the occurrence of heart failure, stroke, coronary heart disease, untreated hypertension, cancer, psychological and neurological or neurodegenerative diseases within the previous three years. For the present analysis, the Short Physical Performance Battery (SPPB; see below) was used to identify functionally fit, robust individuals. Scores of <10 points led to exclusion [27,28]. The study was conducted according to the guidelines laid down in the Declaration of Helsinki and all procedures were approved by the ethics committee of the Friedrich-Alexander-Universität Erlangen-Nürnberg (number: 291_15 B). Written informed consent was obtained from all participants prior to the assessments. The study was registered at the German Clinical Trials Register (DRKS-ID: DRKS00009797). 

**Figure 1:**
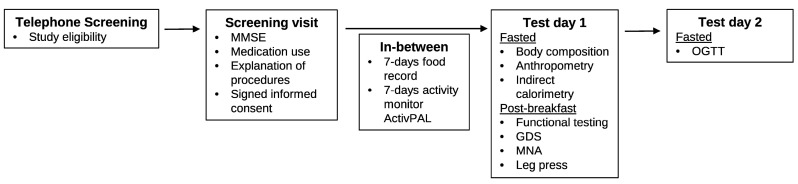
Study procedures. Abbreviations: MMSE: Mini Mental State Examination; GDS: Geriatric Depression Scale; MNA: Mini Nutritional Assessment; OGTT: oral glucose-tolerance test.

An overview of the study procedures is presented in Figure 1. By a systematic telephone interview, general study eligibility was screened and individuals meeting the inclusion criteria were invited to the study center for a screening visit. If eligible, they were scheduled for two test days, each approximately 5 h in length. Participants were asked to refrain from any intense physical activity the evening before and the morning of the two test days. 

### 2.2. Phenotyping of the Participants

The SPPB (0–12 points) including tests for balance (side-by-side, semi-tandem, tandem stances), usual gait speed (4 m course), and functional lower extremity strength (sit-to-stand, 5 repetitions) was applied to identify robust people without functional limitations [27,29]. Each domain scored 0–4 points, with higher scores indicating better performance. An overall sum score was calculated [29]. The SPPB has been shown to have high discriminative, predictive, and concurrent validity as well as excellent reliability [30].

In addition, three subtests of the Fullerton Senior Fitness Test Battery (8-Foot Up-and-Go test, 30 s arm curl test, 6 min walk) were performed [31,32]. The 8-Foot Up-and-Go test was used to assess mobility. The time in seconds required to stand up from a standard chair without arms, walk 8 feet, turn, and return to seated position was recorded. The 30 s arm curl test was performed to assess upper body strength. The number of possible biceps curls holding a dumbbell (women 5 lb; men 8 lb) in 30 s was reported. A 6 min walk test was conducted to assess aerobic endurance. Participants were encouraged to walk a 10 m length course, there and back, as fast as possible. The distance (m) covered within six minutes was measured with an accuracy of 1 m and converted into Yards (yd) for comparison to reference values [31]. The function subscale of the Short-Form Late-Life Function and Disability Instrument (SF-LLFDI; 15–75 points) was used to assess self-rated functional limitations [33]. Higher scores indicate better function. 

The cognitive status was rated with the MMSE (0–30 points) [34]. A score of 24 or more out of 30 possible points indicates the absence of major cognitive impairments. Depressive symptoms were screened using the Geriatric Depression Scale (GDS; 0–15 points) [35]. A score of >7 points indicates depressed mood [36].

The Mini Nutritional Assessment (MNA; 0–30 points) was applied to screen for malnutrition [37]. Participants with 24 to 30 points are considered to be well nourished, a score of 17 to 23.5 points indicates risk of malnutrition, and less than 17 points indicates malnutrition. 

A 75 g oral glucose-tolerance test (OGTT) was performed after overnight fasting. Fasting plasma glucose concentration (mg/dL) and plasma glucose concentration 120 min post glucose intake were immediately analyzed using the HemoCue^®^ Glucose 201+ System (HemoCue, Angelholm, Sweden) and categorized into normal or impaired glucose regulation or type 2 diabetes according to the criteria of The Expert Committee on the Diagnosis and Classification of Diabetes Mellitus [38]. 

Resting energy expenditure (REE, kcal/day) was measured by indirect calorimetry with a Cosmed Quark RMR (Cosmed, Pavona, Italy) by dilution using a canopy [39]. Measurement took place after overnight fasting in a lying position in a separate, quiet room. Measuring duration was 30 min. 

Physical activity and sedentary time were measured using a 7-day (24 h) tri-axial accelerometer (activPAL3™ micro, PAL Technologies Ltd., Glasgow, Scotland, UK) starting at midnight the day after the screening visit and providing information on postural changes in time (h/day) spent sitting/lying (‘sedentary time’), standing/upright, and number of steps per day (‘physical activity’) (activPAL3^TM^ software, version 7.2.32, Glasgow, Scotland, UK). The sensor was placed on the right mid anterior thigh attached using transparent adhesive film. Data were analyzed when a minimum of four complete days were acquired [40]. 

### 2.3. Anthropometry and Body Composition

Body height (in cm, to the nearest 0.1 cm) was measured without shoes using a stadiometer (seca, Hamburg, Germany). BW (in kg, to the nearest 0.05 kg) and body composition by Bioelectrical Impedance Analysis (BIA) were assessed using a Seca medical Body Composition Analyzer 515 (seca, Hamburg, Germany) after overnight fasting. As participants wore light clothes and no shoes, weight was corrected by subtracting 1 kg. Skeletal muscle mass (SMM) was calculated according to the equations of Janssen et al. [41]. SMM was divided by height squared to obtain the skeletal muscle index (SMI, kg/m^2^). An SMI of at most 8.50 kg/m^2^ for men and at most 5.75 kg/m^2^ for women was categorized as ‘low’ [42]. Fat mass (kg) and fat-free mass (kg) were also obtained from BIA. 

### 2.4. Leg Muscle Strength, Leg Muscle Power, and Handgrip Strength

Leg extensor muscle strength and power were measured using a Keiser A420 pneumatic resistance seated leg press machine (Keiser Corporation, Fresno, CA, USA). Firstly, the one-repetition maximum (1-RM, kg) was determined [43]: Testing began with a familiarization warm-up of 10 repetitions with the lightest possible load of 18 kg where proper lifting technique was demonstrated and practiced. Following this, resistance was gradually increased for every single lift aiming to reach the 1 RM in around 6 lifts with at least 2 min of rest between attempts. After a rest of at least 2 min, leg muscle power (W) was measured at eight different percentages of the 1 RM, in ascending order (20%, 30%, 40%, 50%, 60%, 70%, 80%, and 90%) [43,44,45]. Participants were asked to push every load as fast as possible with at least 2 min of rest between attempts. Peak power was identified as the single highest power achieved amongst all eight attempts. Both legs were measured at the same time. Maximum handgrip strength (kg) was measured according to the Southampton protocol for adult grip strength measurement with a Jamar hydraulic hand dynamometer (Sammons Preston Rolyan, Bolingbrook, IL, USA) [46]. The best of the six attempts (three left and three right) was used for analyses. A handgrip strength of <26 kg (men) or <16 kg (women) was categorized as ‘weak’ [47].

### 2.5. Protein Intake

Current dietary intake was assessed using an open 7-day food record. At the screening visit, participants were provided with detailed oral and written instructions by nutritional scientists. All food and beverages, portion sizes (using a scale or household measures), and the time of intake were reported. Participants were instructed to stick to their usual dietary habits. The last reporting day was the day prior to the first test day. On test day 1, the records were checked for completeness and, if necessary, participants were asked for additional information. Food intake was categorized into ‘breakfast’, ‘morning snack’, ‘lunch’, ‘afternoon snack’, ‘dinner’, and ‘evening snack’ based on time of intake and size/composition of meal. Energy, macronutrient, and leucine intake was calculated per day and per meal using EBISpro software (EBISpro, Willstätt-Legelshurst, Germany, 2016) based on the German nutrient database ‘Bundeslebensmittelschlüssel’ (version 3.02, Karlsruhe, Germany) [48]. Data entry was checked by a second nutritional scientist. 

The evenness of protein distribution across the three main meals was expressed by the dimensionless coefficient of variation (CV) of protein intake at breakfast, lunch, and dinner (CV = standard deviation/mean)—the smaller the CV, the more even the distribution. Protein intake per day and per meal in g was divided by participant’s BW in kg to obtain the relative intake. Number of meals per day containing ≥0.4 g protein/kg BW and ≥2.5 g leucine, respectively, were counted with a potential maximum number of six meals. In addition, to determine the potential accumulative importance of the four aspects, a composite score of protein intake pattern (daily amount, evenness of distribution, number of meals providing ≥0.4 g protein/kg BW, number of meals providing ≥2.5 g leucine) was calculated for each of the seven days. The highest possible score that could be reached per day was 4 points, reflecting an intake ≥1.0 g protein/kg BW/day, plus a relatively even distribution of protein at breakfast, lunch, and dinner (CV < 0.44; lowest tertile of the mean CV of seven days in the total sample), plus at least two meals containing ≥0.4 g protein/kg BW, plus at least two meals containing ≥2.5 g leucine. The lowest score was 0 points for those not meeting any aspect. Detailed information on categorization of the scoring can be found in Table 1. For all five protein intake variables, the mean of the seven days was used for analyses. 

### 2.6. Data Analysis and Statistics

**Figure 2:**
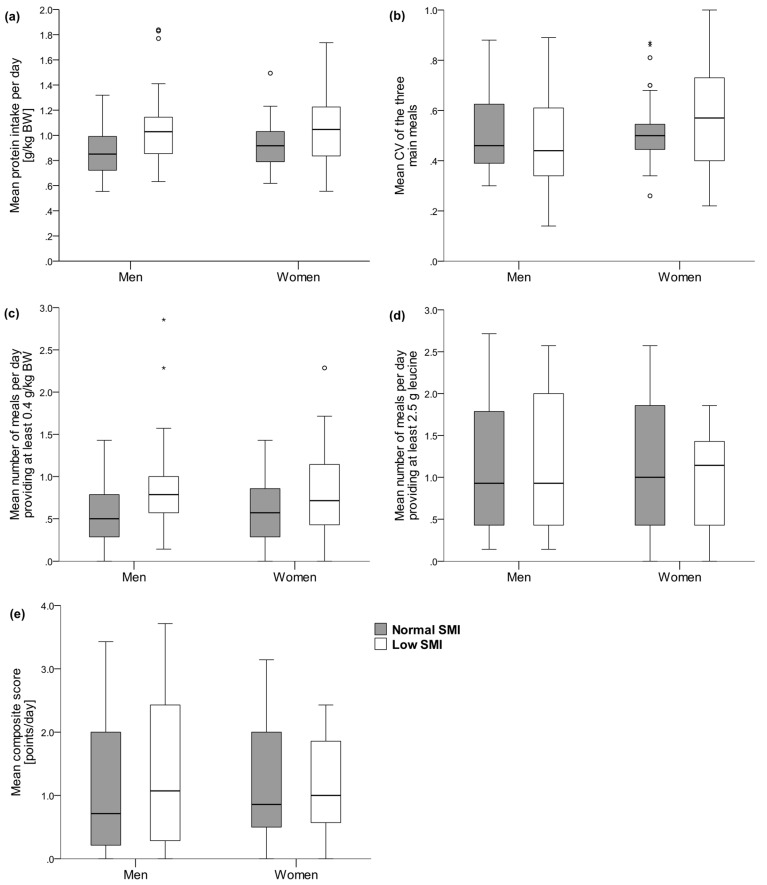
Mean daily intake of protein (**a**), mean coefficient of variance across main meals (**b**), mean number of meals providing at least 0.4 g protein/kg BW per day (**c**), mean number of meals providing at least 2.5 g leucine per day (**d**) and mean composite score representing protein intake pattern (**e**) obtained from 7-day food records in healthy community-dwelling older men with normal (*n* = 30) and low (*n* = 19) skeletal muscle index (SMI) and in women with normal (*n* = 31) and low (*n* = 17) SMI. Abbreviation: CV: coefficient of variance of protein intake (g) at breakfast, lunch, and dinner. The composite score combines all four aspects of protein intake: daily amount, number of meals providing ≥0.4 g/kg BW protein, number of meals providing ≥2.5 g leucine, and evenness of protein distribution across breakfast, lunch, and dinner. Score range: 0–4 points per day, best: 4 points. All *p* > 0.05, Mann–Whitney U test. The boxes represent interquartile ranges with the bold horizontal lines denoting the median. The whiskers show the highest and lowest values within the 1.5-fold interquartile range. The circles represent outliers and asterisks represent extreme outliers.

Statistical analyses were performed with SPSS Version 24 (IBM SPSS Statistics, Chicago, IL, USA). All data were analyzed separately for men and women. Descriptive statistics were used for participants’ characteristics and dietary intake data. Data were tested for normality using the Shapiro–Wilk test. Participants’ characteristics (Table 2) are presented as mean ± standard deviation (SD) (normally distributed) or median and interquartile range (IQR). All dietary intake data (Table 3) are presented as mean ± SD. Differences in continuous data between men and women were tested using the independent samples *t*-test (normally distributed) or the Mann–Whitney U test. For categorical data, the Chi^2^ test was applied. Bivariate association between aspects of protein intake and composite score and SMI, leg muscle strength, leg muscle power, and handgrip strength were examined using Spearman rank correlation coefficients (Table 4). To test for differences in aspects of the protein intake as well as composite score between participants with normal and low SMI, the Mann–Whitney U test was used (Figure 2). The level of significance was set at *p* < 0.05. 

## 3. Results

### 3.1. Characteristics of the Study Participants

Two hundred and sixty individuals were screened for eligibility by structured telephone calls. One hundred and fifty-nine individuals did not fulfill inclusion criteria. Of 101 eligible and tested individuals, three participants were excluded from the analysis because of missing leg press tests (dependent variable) and one man was excluded because of a mean energy intake of more than 3 SD from the mean. In total, 97 participants were included in the analyses. Participants’ characteristics are presented in Table 2. Median age was 77 (76.0–80.5) years with no difference between men and women. The median SPPB score was 12.0 (12.0–12.0) points, demonstrating the robustness of the sample. No differences in the 6 min walk, 8-Foot Up-and-Go test, and arm curl test were observed between men and women. The LLFDI function subscale was lower in women (68.5 (61.5–71.0)) compared with men (72.0 (66.0–73.0)) (*p* = 0.002). Women spent significantly less time sitting or lying down compared with men (*p* = 0.014), although the number of steps per day was not different between the sexes (*p* = 0.251). A total of 95.9% of participants had a normal nutritional status according to MNA, and none were categorized as malnourished. Diabetes was found in three men and in no women, and glucose regulation was impaired in 34.7% of men and 25.5% of women (*p* = 0.061).

### 3.2. Muscle Mass and Muscle Strength

SMM, SMI, fat free mass, leg muscle power, leg muscle strength, and handgrip strength were significantly lower in women than in men (*p* < 0.001). A low SMI was found in 38.8% of men and 35.4% of women (*p* = 0.732). Muscle weakness, as measured by handgrip strength, was observed in two women (4.2%) and in two men (4.1%) (*p* = 0.983). 

### 3.3. Aspects of Protein Intake

Dietary intake data are presented in Table 3. In total, 38.8% of men and 39.6% of women consumed an average of 1.0 g protein/kg BW or more. At least 1.0 g/kg BW/d was reached on each day of the week in 10.2% of men and 4.2% of women, whereas 22.4% of men and 20.8% of women did not reach this amount at any day of the week (*p* = 0.425). 

Protein intake was highest at lunch, closely followed by dinner. The least protein was eaten at breakfast. The CV ranged from 0.14 to 1.10. An ‘even’ distribution (CV < 0.44) on every day of the week was found in 4.1% of men and 4.2% of women, whereas protein intake was ‘uneven’ (CV ≥ 0.44) on any day in 18.4% of men and 14.6% of women (*p* = 0.545). 

The mean number of daily meals providing at least 0.4 g protein/kg BW ranged from 0 to 2.86, while 4.1% of men and no women consumed two or more meals per day providing at least 0.4 g protein/kg BW on each of the seven days. Of the studied population, 57.1% of men and 52.1% of women did not reach two or more meals with ≥0.4 g protein/kg BW on any day of the week (*p* = 0.719). 

The mean number of meals providing at least 2.5 g leucine ranged from 0 to 2.71 per day. A total of 14.3% of men and 4.2% of women reached two meals providing 2.5 g leucine every day, whereas 34.7% of men and 31.3% of women did not reach two meals on any day of the week. 

The protein intake composite score ranged from 0 to 3.71 points per day (mean over seven days). The best possible composite score of 4 points meeting all four suggestions was reached at least three times per week by 10.2% of men and no women, while 63.3% of men and 58.3% of women did not reach 4 points on any day. Of the studied population, 14.3% of men and 14.6% of women had a composite score of 0 points not meeting any of the four suggestions on all of the seven days. 

### 3.4. Association between Aspects of Protein Intake and Muscle Mass and Strength

In Table 4, the binary correlation coefficients between all four aspects of protein intake as well as the composite score and SMI, leg muscle power, leg muscle strength, and handgrip strength are presented. In men, a weak correlation between the mean CV and SMI (*p* = 0.043) was found, indicating an inverse association between evenness of protein intake and muscle mass. No other significant correlations were found. Scatterplots for these associations can be found in Supplementary File 1. 

In Figure 2, all four aspects of protein intake (a–d) as well as the composite score (e) are displayed for men and women stratified by low and normal SMI. No significant differences between those with normal SMI and those with low SMI were observed. 

## 4. Discussion

This study aimed to test the hypothesis of whether single and combined adherence to previously published suggestions for optimal protein intake is associated with muscle mass, strength, and power among healthy community-dwelling adults without functional limitations aged 75–85 years. Contrary to our initial hypothesis, we found no positive association between relative protein intake, the evenness of protein distribution, number of meals providing at least 0.4 g protein/kg BW or 2.5 g leucine or their combination and SMI, leg muscle power, leg muscle strength, or handgrip strength. 

The participants included were successful agers, which is reflected in their fast gait speed, low self-rated functional limitations, good cognitive status, good emotional status without depressive symptoms, and normal nutritional status (Table 2). Individuals were also 20% more physically active and 5% less sedentary than a sample of community-dwelling adults with a similar mean age of 78 years using the same method of data acquisition [49]. With regard to the positive effects of physical activity on functional outcomes described in a systematic review [50], this difference appears to be relevant. 

In this sample of successful agers, the adherence to the four aspects of ideal protein intake pattern was moderate to low. Mean protein intake was 1.0 g/kg BW/day, which is similar to other studies [12,14,19,20,21]. The lowest adherence was observed for the 0.4 g protein/kg BW/meal threshold: Less than one meal providing at least 0.4 g protein/kg BW was consumed on average per day and only six participants (6.2%) reached three meals with 0.4 g protein/kg BW per day at least once in a week. This confirms findings from Cardon-Thomas et al., the first study looking at the 0.4 g protein/kg threshold in older adults (mean age 78 years) in the UK [49]. They found that 8% of participants did not reach the threshold for any of their daily meals and no participant consumed three meals per day reaching the threshold, although all protein intake occasions (including snacks) were, based on time periods, categorized into breakfast, lunch, or dinner [49]. Due to the low adherence to the 0.4 g protein/kg BW threshold, for the composite score, the number of meals providing adequate protein or leucine were categorized ≥2 instead of ≥3 meals/day [7,9]. Protein intake in our sample was more evenly distributed across the main meals, represented by a CV of 0.53 ± 0.19 compared with 0.67 ± 0.2 in the study by Cardon-Thomas et al. [49]. The daily leucine intake of 7.80 g in our female subsample is higher than in previous observations of 5.68 g/day in non-frail community-dwelling Japanese women with a mean age of 75 years [51].

Contradicting results between our study not finding any association and some previous studies observing associations between daily amount of protein, evenness of protein intake, or number of meals providing adequate protein and muscle mass, strength, or function are likely in part due to differences in study populations. To our knowledge, this is the first study examining these associations in successful agers. Our results question the relevance of these suggestions for this subgroup. Previous studies investigated rather young populations [12,13,18,19,21] for which anabolic resistance might be at an earlier stage. Other observations among heterogeneous populations represented a broad range of functional status [14,20,51]. This heterogeneity may be needed to identify associations between protein intake characteristics and parameters of muscle mass and function. 

Using the cut points by Janssen et al. [42], muscle mass in our study sample was low in 38.8% of men and 35.4% of women. In the original NHANES analyses, the proportion of low SMI was 11% in men and 9% in women [42]. However, in these analyses, participants aged 60 and older were included and, considering the progressive age-related loss of fat-free mass [52], a higher proportion in our study is reasonable. Also, differences in measurement methods (conventional wrist–ankle BIA vs. 8-point segmental system) have to be considered [53]. However, it appears that the lower muscle mass did not translate into reduced strength. Only two men and two women (approximately 4%) were weak as measured by handgrip strength. Especially for women, this proportion is considerably lower than reported by large cohort studies [47]. 

Finding no association between protein intake and muscle mass in our rather healthy older population is in line with results by Houston et al. [11]. In their study, no cross-sectional differences in (appendicular) lean mass between community-dwelling adults aged ~74 years consuming 0.8 vs. 1.2 g protein/kg BW/day were found [11]. Moreover, Scott et al. did not observe differences in muscle strength in community-dwelling adults aged ~62 years between those meeting the recommended dietary allowance (RDA) and those not, although they showed an association between protein intake and appendicular lean mass [13]. Likewise, no differences in handgrip strength were found comparing healthy community-dwelling older women consuming protein above the RDA to those below the RDA [54]. In contrast, the Women’s Health Initiative with a mean age below 70 years found a small—but significant—difference in grip strength of 0.6 kg between the highest (1.19 g/kg BW/day) and lowest quintile of protein intake (0.97 g/kg BW/day) [12]. Compared to our study, protein intake of the lowest quintile was similar to the mean intake in the current sample. Yet, when looking at the quintile with the highest handgrip strength, the mean age at follow-up was 11 years younger, but mean handgrip strength was lower than that of women in our study. In another study, conducted in functionally limited (mean SPPB < 7 points) women with a mean age of 68 years, protein intake was associated with gait speed, one-leg stance duration, and muscle strength [14]. These results may indicate that protein intake is associated with measures of muscle mass, strength, and physical performance in individuals with functional limitations but not in healthy unimpaired older adults. 

We observed a large variability and heterogeneity in the distribution of protein intake across the main meals. Some individuals ate nearly equal amounts of protein at breakfast, lunch, and dinner (e.g., 36.8 g, 36.2 g, and 38.9 g) while others consumed protein in a skewed manner (e.g., 10.2 g, 8.8 g, and 25.2 g; or 11.7 g, 46.9 g, and 4.3 g). Nonetheless, no positive association between the evenness of protein intake and muscle mass, strength, or power was observed. Contrary, a weak—but significant—negative correlation between the evenness of protein intake and SMI was present in men. However, when excluding the three men with diabetes mellitus from the analyses, as diabetes is known to affect muscle metabolism [55], this weak correlation was no longer significant (*p* = 0.090). Therefore, we interpret this weak correlation as not clinically meaningful. In a previous cross-sectional study, associations of even protein intake with lean mass were observed in men, but not in women [19] and with muscle strength in women but not in men, none of which could be confirmed in our sample [18]. Bollwein et al. observed a more even protein intake pattern in robust older adults compared with frail persons [20]. Supporting this, in our sample of successful agers, a lower median CV (0.50) compared with non-frail persons (0.68) in this previous study was observed [20]. Moreover, experimental studies have shown inconsistencies in their results regarding the protein intake distribution pattern which may be caused by the age-associated anabolic resistance: Whereas in younger adults (age ~37 years), higher MPS rates were observed when protein was evenly distributed in a 7-day feeding study [15], in older adults, Kim et al. found no differences between even and skewed protein intake in protein kinetics at whole body or muscle level in a 4-day feeding study (mean age > 64 years) [17] or in lean mass, muscle strength, or whole-body protein kinetics in an 8-week intervention study (age ~59 years) [16]. However, the authors argue that the protein intake per meal of 0.37 g/kg BW in the even pattern might have been insufficient to reach the anabolic per-meal threshold in the 8-week intervention [16]. 

To date, the only epidemiological study on the relation between per-meal protein amount and lean mass and strength is the cross-sectional analysis by Loenneke et al. using NHANES data. In this representative sample of US adults with a mean age of 60 years, the number of meals per day with at least 30 g of protein was associated with leg lean mass and muscle strength [21]. In our sample, no relationship between the number of meals providing adequate protein and muscle mass, strength, or power was observed, neither for the 0.4 g protein/kg BW (Table 4, Figure 2) nor the 30 g per meal approach (data not shown). Both studies have a low adherence to the per-meal concept in common: In the study of Loenneke et al., only approximately 16% of participants ate at least two meals per day with 30 g of protein [21]. However, we consider the relative to BW approach as more reasonable since protein requirements depend on body mass. The threshold of 0.4 g protein/kg BW derives from studies in healthy men (mean age ~71 years) investigating high quality, rapidly digestible, animal-based protein under laboratory conditions [3]. Hence, in the present study, when protein was consumed as mixed meals under real-life circumstances, the critical amount of protein required to maximally stimulate MPS might be even higher [56]. However, the high level of physical activity found in our sample may act as an anabolic stimulus and counteract anabolic resistance [57,58]. Furthermore, differences in post-absorptive muscle metabolism between men and women need to be considered [59,60]. Future experimental studies should investigate the protein threshold to maximally stimulate MPS and to suppress protein breakdown [61] under real-life circumstances (physical activity; co-ingestion of other dietary components, such as macronutrients and dietary fibre; sex-specificity) to provide the rationale for adequate and differentiated dietary guidelines. 

Furthermore, no association between the number of meals providing adequate leucine and muscle mass, strength, or power was found (Table 4, Figure 2). In accordance with this finding, a three-month supplementation of 2.5 g leucine with each main meal did not improve muscle mass or strength in healthy older men consuming adequate protein (approximately 1.0 g/kg BW/day) [62]. Future epidemiological research is warranted to confirm our findings and to examine whether this threshold is clinically relevant in other samples. 

In addition to the analysis of the four single aspects of protein intake, we constructed a composite score to determine the potential relevance of accumulating the four dimensions. The aspects were equally weighted. Whether particular aspects are more important than others in terms of preservation of muscle mass and function has not yet been established and requires further research. The large range in sum scores from 0 to 3.71 points per day reflects the diversity in protein intake patterns. However, as for the single aspects of protein intake, no relationship between the composite score and muscle mass, strength, or power was found. One could argue that, in terms of muscle mass, strength, or power, successful agers do not benefit from greater intake of high-quality protein (total and per-meal) and a more even distribution of protein intake. However, additional studies are needed to clarify this question. In agreement with this hypothesis, a recent meta-analysis found no evidence that protein supplementation increases muscle mass or strength in healthy older people without concomitant exercise intervention [63]. In healthy older people with good functional status, other factors may be more crucial for maintaining muscle mass and strength than protein intake as long as it is within an adequate range. These factors may include routine resistance exercise training [64], regular physical activity, and reduced sedentary time [5], as well as muscle mass and resistance exercise training in earlier life stages [64]. 

A strength of the present study is the comprehensive clinical phenotyping of this healthy study population, allowing generalizability of the findings on this particular subpopulation. Secondly, the investigation of all four previously published suggestions of protein intake within one study sample is unique. Thirdly, by using objective continuous performance-based test measures (handgrip strength, leg muscle strength and power), we were able to distinguish the performance of the participants and to avoid ceiling effects. In particular, muscle power is a critical and sensitive determinant of physical function since it declines earlier and at a higher rate and correlates better with functional parameters than muscle strength [2]. Fourthly, dietary intake was prospectively assessed using 7-day food records. In order to minimize the risk of underreporting, participants were instructed to stick to their usual diet, every protocol was thoroughly checked for completeness, and missing information was immediately obtained. The 7-day food record may offer some advantages, as it allows assessment and analysis of variations in protein intake across a full 7-day period. For instance, on Sunday, the suggestion of two meals providing at least 0.4 g protein/kg BW was more frequently reached (21.6%) than on other days (data not shown). However, it should be kept in mind that this method does not assess usual dietary intake and is, as all dietary assessment instruments, restricted to participants’ self-report. We also acknowledge several limitations. Firstly, the sample size of 97 participants was rather small and the data are not normally distributed. Besides non-significant results, the levels of correlation were small, suggesting that an increase in sample size is unlikely to change this finding. Secondly, due to the cross-sectional design, causal relationships cannot be derived. Thirdly, even though physician-diagnosed diabetes was an exclusion criterion, diabetes was found in three participants (6.1%). Fourthly, body composition, including muscle mass, was assessed using BIA, which might limit the comparability to studies using other methods, e.g., DXA, and is often considered less precise. However, we used an age-specific, magnetic resonance imaging-based BIA equation to calculate muscle mass [41] and applied specific BIA cut-offs that have been validated in large-scale epidemiological studies [42]. Finally, the per-meal concept suggested by Murphy et al. [9] is not fulfilled in our study. Thus, it remains a matter of future research to recruit healthy community-dwelling older adults habitually meeting this recommendation and to examine whether associations with muscle mass, strength, or performance exist.

## 5. Conclusions

Neither one of the single suggestions on protein intake for maintenance of muscle mass and function for healthy older adults, nor their combination, were associated with muscle mass, strength, or power in this sample of healthy community-dwelling older adults aged 75–85 years. These results question the relevance of these suggestions for successful agers without functional limitations and with a protein intake within the usual range. Additional epidemiological and experimental studies are needed investigating the impact of meeting all protein intake suggestions on a daily basis on muscle mass and function. Such studies should consider the age and functional status of the participants.

## Figures and Tables

**Table 1:** Categorization of the protein intake composite score.

	Points/Day	Categorization
Protein amount	0	<1.0 g/kg BW/day
	1	≥1.0 g/kg BW/day
Evenness of protein distribution across the three main meals	0	CV ≥ 0.44
	1	CV < 0.44
Number of meals providing ≥0.4 g protein/kg BW	0	0 or 1 meal/day
	1	≥2 meals/day
Number of meals providing ≥2.5 g leucine	0	0 or 1 meal/day
	1	≥2 meals/day
Composite score (sum)	0	Minimum score
	4	Maximum score

The cut-off value of the coefficient of variance (CV) is the lowest tertile of the mean CV of seven days in the total sample. BW = body weight.

**Table 2:** Characteristics of the total healthy community-dwelling sample of older adults and subgroups of male and female participants (median (IQR) or mean ± standard deviation).

	All (*n* = 97)	Men (*n* = 49)	Women (*n* = 48)
**General Characteristics**			
Age (years)	77.0 (76.0–80.5)	78.0 (76.0–80.0)	77.0 (76.0–81.0)
MMSE (points)	29.0 (28.0–30.0)	29.0 (28.5–30.0)	29.0 (28.0–30.0)
GDS (points)	1.0 (0.0–2.0)	1.0 (0.0–2.0)	1.0 (0.0–2.0)
MNA (points)	27.5 (26.0–28.5)	27.5 (26.0–28.5)	27.5 (26.1–28.5)
Body weight (kg)	74.1 ± 14.0	81.9 ± 11.9	66.0 ± 11.1 *
BMI (kg/m^2^)	26.8 ± 4.0	27.3 ± 3.7	26.2 ± 4.3
**Body Composition, Muscle Power, and Muscle Strength**			
Fat mass (kg)	28.1 ± 7.7	26.9 ± 7.8	29.4 ± 7.5
Fat free mas (kg)	47.5 (36.6–55.3)	55.3 (51.6–60.3)	36.6 (33.2–41.8) *
Skeletal muscle mass (kg)	21.3 (15.4–26.1)	26.0 (24.6–28.1)	15.4 (13.9–17.5) *
Skeletal muscle index (kg/m^2^)	7.5 (6.1–8.8)	8.8 (8.3–9.2)	6.1 (5.6–6.6) *
Max. leg muscle power (W)	290.5 (244.3–410.6)	396.3 (313.4–545.9)	245.5 (177.6–282.8) *
Max. leg muscle strength (kg)	185.0 (150.0–250.0)	250.0 (205.0–265.0)	150.0 (120.0–163.8) *
Max. handgrip strength (kg)	30.0 (22.0–38.0)	38.0 (34.5–41.0)	22.5 (19.0–26.0) *
30 s arm curl test (repetitions)	16.0 (15.0–19.0)	16.0 (14.5–19.0)	16.0 (15.0–18.8)
**Physical Performance and Function**			
6 min walk (yd)	494.8 ± 70.7	502.1 ± 81.0	487.4 ± 58.3
8-Foot Up-and-Go (s)	5.8 (5.2–6.4)	5.6 (5.1–6.7)	5.9 (5.3–6.3)
4 m gait speed (m/s)	1.3 (1.1–1.4)	1.3 (1.1–1.4)	1.2 (1.1–1.3)
LLFDI-function subscale	70.0 (65.0–73.0)	72.0 (66.0–73.0)	68.5 (61.5–71.0) *
**Factors Influencing Muscle Metabolism**			
REE (kcal/day)	1524.4 ± 284.0	1706.3 ± 239.9	1338.8 ± 190.6 *
Physical activity (steps/day) ^b^	9071.5 (7874.5–11283.0) ^a^	9335.0 (8210.5–12177.0)	8923.0 (7718.0–10886.0) ^a^
Sedentary time (h sitting or lying/day) ^b^	17.2 ± 1.6 ^a^	17.6 ± 1.6	16.8 ± 1.6 ^a,^*
Fasting plasma glucose (mg/dL)	91.5 (84.0–100.0) ^a^	97.0 (84.0–102.0)	88.0 (84.0–97.0) ^a^
OGTT: Plasma glucose at 120 min (mg/dL)	99.0 (80.3–117.5) ^a^	94.0 (78.0–110.5)	105.0 (91.0–120.0) ^a^

^a^ *n* = 1 missing. ^b^ activPAL activity monitor with ≥4 days of complete recording. Abbreviations: IQR: interquartile range; MMSE: Mini Mental State Examination (score range: 0–30); GDS: Geriatric Depression Scale (score range: 0–15); MNA: Mini Nutritional Assessment (score range: 0–30); yd: Yards; LLFDI: Late-Life Function and Disability Instrument (score range: 15–75 points); REE: resting energy expenditure; OGTT: oral glucose-tolerance test. * *p* < 0.05, *t*-test or Mann–Whitney U test.

**Table 3:** Daily mean dietary intake data obtained from 7-day food records of the total healthy community-dwelling sample of older adults and subgroups of male and female participants (mean ± standard deviation).

	All (*n* = 97)	Men (*n* = 49)	Women (*n* = 48)
**Energy and Macronutrient Intake Per Day**			
Energy ^a^ (kcal)	1912 ± 442	2115 ± 453	1705 ± 320 #
Carbohydrate ^a^ (g)	200.7 ± 58.5	222.0 ± 64.6	178.9 ± 41.9 #
Fat ^a^ (g)	81.5 ± 20.1	86.7 ± 22.1	76.1 ± 16.5 #
Protein ^a^ (g)	70.1 ± 18.9	77.2 ± 20.8	62.9 ± 13.6 #
Protein (Energy %)	15.2 ± 2.8	15.0 ± 2.7	15.3 ± 2.8
Protein ^a^ (g/kg BW)	0.97 ± 0.28	0.96 ± 0.30	0.97 ± 0.25
**Absolute Protein Intake Per Meal**			
Protein breakfast ^a^ (g)	16.45 ± 8.58	17.71 ± 8.73	15.15 ± 8.30
Protein lunch (g)	24.18 ± 9.92	26.36 ± 10.02	21.96 ± 9.42 *
Protein dinner ^a^ (g)	21.86 ± 10.45	25.44 ± 11.19	18.20 ± 8.27 #
CV ^a^	0.53 ± 0.19	0.51 ± 0.20	0.55 ± 0.17
**Relative Protein Intake Per Meal**			
Protein breakfast ^a^ (g/kg BW)	0.23 ± 0.12	0.22 ± 0.11	0.23 ± 0.13
Protein morning snack ^a^ (g/kg BW)	0.02 ± 0.04	0.02 ± 0.03	0.02 ± 0.04
Protein lunch (g/kg BW)	0.33 ± 0.14	0.33 ± 0.13	0.34 ± 0.15
Protein afternoon snack ^a^ (g/kg BW)	0.06 ± 0.04	0.05 ± 0.04	0.07 ± 0.04 #
Protein dinner ^a^ (g/kg BW)	0.30 ± 0.14	0.32 ± 0.15	0.28 ± 0.13
Protein evening snack ^a^ (g/kg BW)	0.02 ± 0.02	0.02 ± 0.02	0.02 ± 0.03
Number of meals providing ≥0.4 g/kg BW per day ^a^	0.72 ± 0.50	0.73 ± 0.54	0.71 ± 0.47
**Leucine Intake Per Day and Per Meal**			
Total daily leucine intake ^a^ (g)	7.92 ± 3.46	8.04 ± 3.59	7.80 ± 3.36
Leucine breakfast (g)	1.97 ± 1.31	2.07 ± 1.48	1.86 ± 1.13
Leucine morning snack ^a^ (g)	0.21 ± 0.43	0.17 ± 0.27	0.25 ± 0.54
Leucine lunch ^a^ (g)	2.50 ± 1.27	2.58 ± 1.24	2.42 ± 1.31
Leucine afternoon snack ^a^ (g)	0.59 ± 0.49	0.48 ± 0.38	0.70 ± 0.57
Leucine dinner ^a^ (g)	2.27 ± 1.26	2.48 ± 1.23	2.06 ± 1.26
Leucine evening snack ^a^ (g)	0.38 ± 0.62	0.25 ± 0.36	0.51 ± 0.79
Number of meals providing ≥2.5 g leucine per day ^a^	1.11 ± 0.76	1.18 ± 0.78	1.04 ± 0.74
**Composite Score ^a^**			
Points/day	1.19 ± 0.96	1.24 ± 1.06	1.14 ± 0.86
Points/day (median (IQR))	1.00 (0.36–2.14)	1.00 (0.29–2.14)	1.00 (0.46–2.07)

^a^ Not normally distributed. The composite score combines four aspects of protein intake: daily amount, number of meals providing ≥0.4 g protein/kg BW, number of meals providing ≥2.5 g leucine, and evenness of protein distribution across breakfast, lunch, and dinner. Score range: 0–4 points per day, best: 4 points. Abbreviations: BW: body weight; CV: coefficient of variance of protein intake (g) at breakfast, lunch, and dinner; IQR: interquartile range. * *p* < 0.05, *t*-test. # *p* < 0.05, Mann–Whitney U test.

**Table 4:** Spearman correlation coefficients between mean daily intake of protein, mean coefficient of variance of protein across main meals, mean number of meals providing at least 0.4 g protein/kg BW, mean number of meals providing at least 2.5 g leucine, and mean composite score representing protein intake pattern obtained from 7-day food records and muscle mass, leg muscle power, leg muscle strength, and handgrip strength in healthy community-dwelling older adults and subgroups of male and female participants.

	Skeletal Muscle Index (kg/m^2^)			Maximum Leg Muscle Power (W)			Maximum Leg Muscle Strength (kg)			Maximum Handgrip Strength (kg)		
	All	Men	Women	All	Men	Women	All	Men	Women	All	Men	Women
	(*n* = 97)	(*n* = 49)	(*n* = 48)	(*n* = 97)	(*n* = 49)	(*n* = 48)	(*n* = 97)	(*n* = 49)	(*n* = 48)	(*n* = 97)	(*n* = 49)	(*n* = 48)
Protein intake per day (g/kg BW)	−0.163	−0.227	−0.143	−0.110	−0.087	−0.060	−0.153	−0.035	−0.253	−0.080	−0.022	0.005
CV of protein intake at main meals	−0.019	0.291 *	0.046	0.020	0.197	0.013	−0.044	0.145	0.001	−0.060	0.104	−0.018
Number of meals per day providing ≥0.4 g protein/kg BW	−0.082	−0.225	−0.021	−0.124	−0.167	−0.100	−0.098	0.030	−0.269	−0.061	−0.118	0.013
Number of meals per day providing ≥2.5 g leucine	0.075	−0.144	0.151	0.011	−0.122	0.047	0.031	−0.074	−0.047	−0.016	−0.132	−0.108
Composite score (points/day)	−0.044	−0.280	0.071	−0.084	−0.199	−0.017	−0.065	−0.130	−0.115	−0.078	−0.145	−0.098

**Abbreviation:** CV: coefficient of variance of protein intake (g) at breakfast, lunch, and dinner. The composite score combines all four aspects of protein intake: daily amount, number of meals providing ≥0.4 g/kg BW protein, number of meals providing ≥2.5 g leucine, and evenness of protein distribution across breakfast, lunch, and dinner. Score range: 0–4 points per day, best: 4 points. * *p* = 0.043; all other cells *p* > 0.05.

## Supplementary Materials

The following are available online at www.mdpi.com/2072-6643/9/12/1358/s1, Figure S1: Scatterplots of association of mean daily intake of protein per kg body weight (BW) and muscle mass, leg muscle power, leg muscle strength and handgrip strength in healthy community-dwelling older men (*n* = 49) and women (*n* = 48); Figure S2: Scatterplots of association of the mean coefficient of variance of protein across main meals and muscle mass, leg muscle power, leg muscle strength and handgrip strength in healthy community-dwelling older men (*n* = 49) and women (*n* = 48); Figure S3: Scatterplots of association of mean number of meals providing at least 0.4 g protein/kg body weight (BW) and muscle mass, leg muscle power, leg muscle strength and handgrip strength in healthy community-dwelling older men (*n* = 49) and women (*n* = 48); Figure S4: Scatterplots of association mean number of meals providing at least 2.5 g leucine and muscle mass, leg muscle power, leg muscle strength and handgrip strength in healthy community-dwelling older men (*n* = 49) and women (*n* = 48); Figure S5: Scatterplots of association of mean composite score representing protein intake pattern and muscle mass, leg muscle power, leg muscle strength and handgrip strength in healthy community-dwelling older men (*n* = 49) and women (*n* = 48).
