# Supplementary material for: Amount, Distribution, and Quality of Protein Intake Are Not Associated with Muscle Mass, Strength, and Power in Healthy Older Adults without Functional Limitations—An enable Study
Source: Nutrients. 2017 Dec 14;9(12):1358. doi: 10.3390/nu9121358 (PMC5748808; doi:10.3390/nu9121358)
Supplement: Supplementary file 1 [file nutrients-09-01358-s001.pdf]

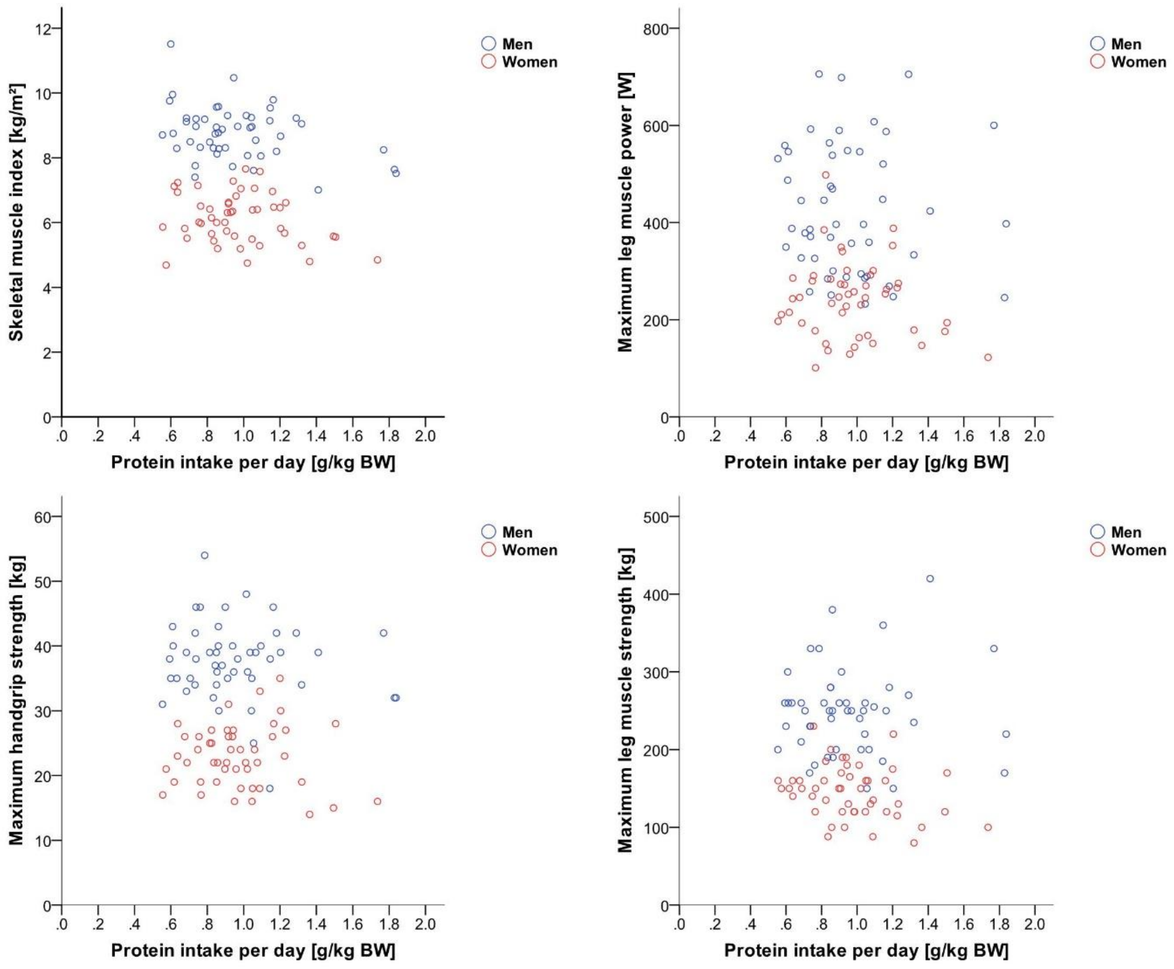

Supplementary figure S1: Scatterplots of association of mean daily intake of protein per kg body weight (BW) and muscle mass, leg muscle power, leg muscle strength and handgrip strength in healthy community-dwelling older men (n = 49) and women (n = 48).

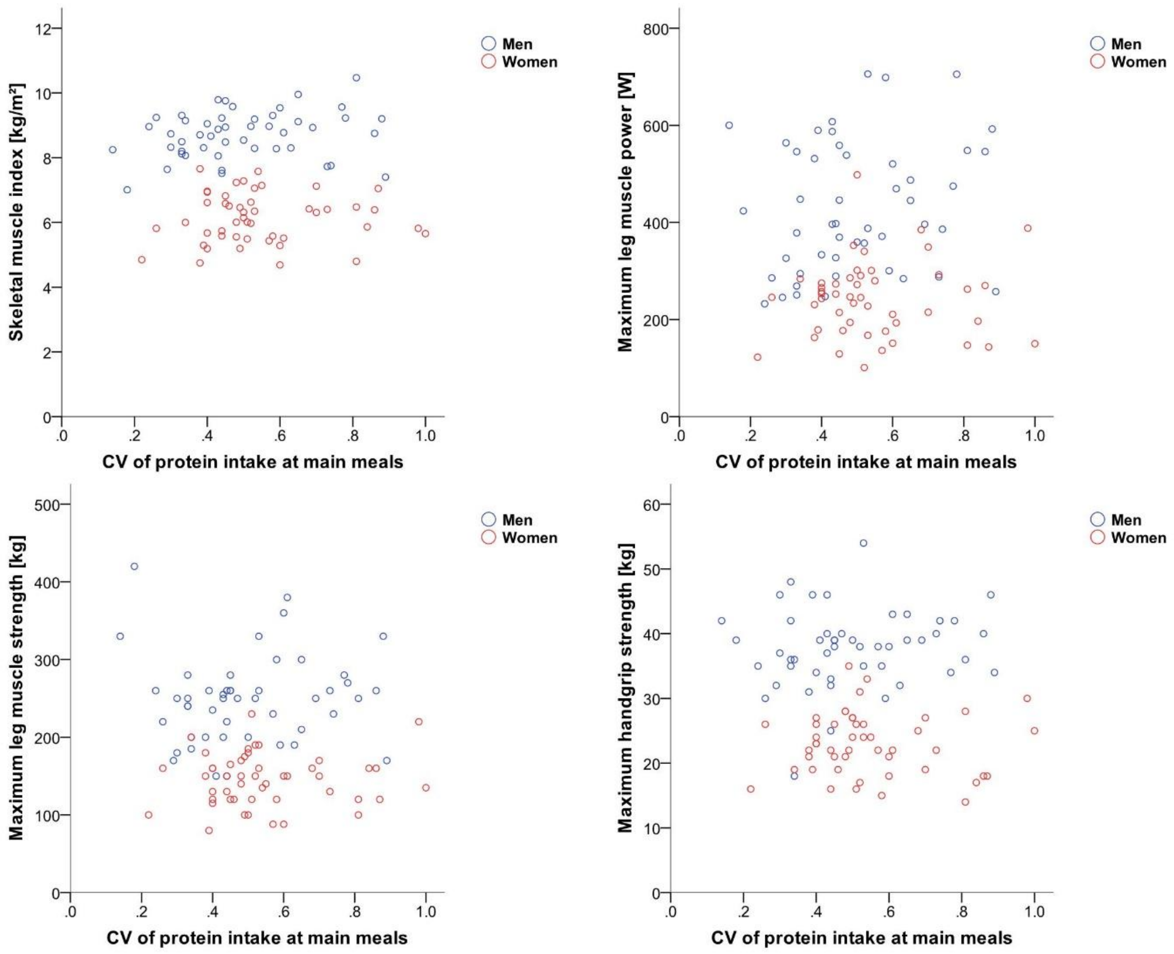

Supplementary figure S2: Scatterplots of association of the mean coefficient of variance of protein across main meals and muscle mass, leg muscle power, leg muscle strength and handgrip strength in healthy community-dwelling older men (n = 49) and women (n = 48).

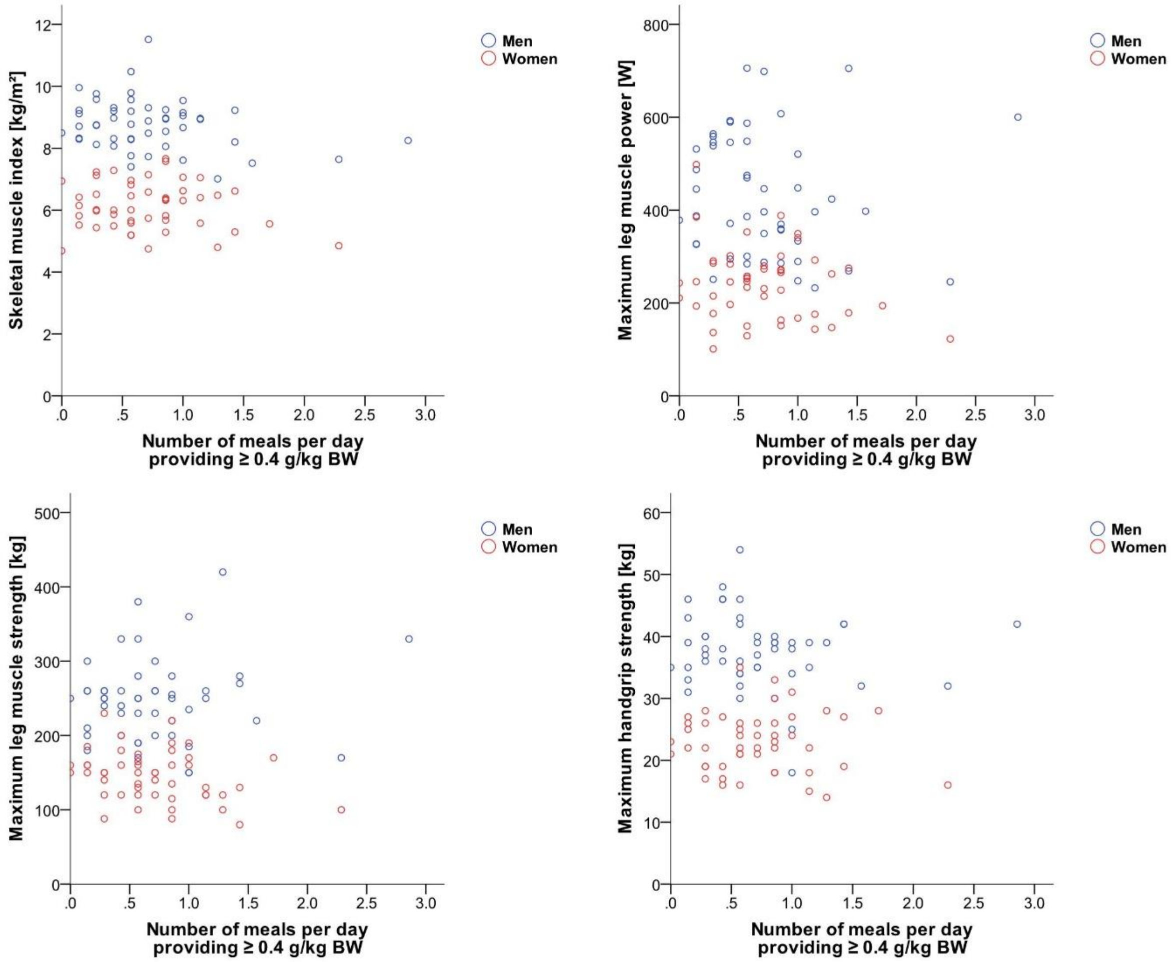

Supplementary figure S3: Scatterplots of association of mean number of meals providing at least 0.4 g protein/kg body weight (BW) and muscle mass, leg muscle power, leg muscle strength and handgrip strength in healthy community-dwelling older men (n = 49) and women (n = 48).

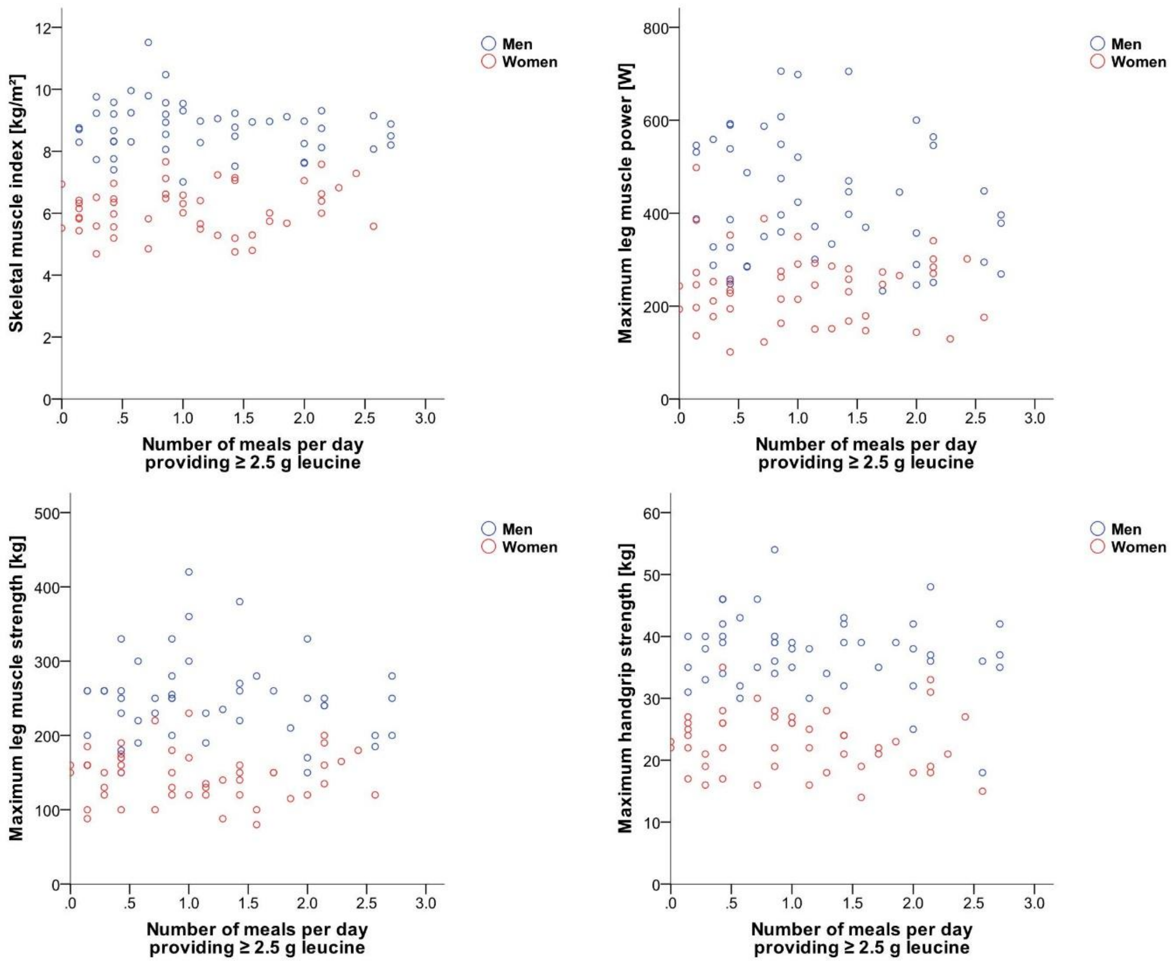

Supplementary figure S4: Scatterplots of association mean number of meals providing at least 2.5 g leucine and muscle mass, leg muscle power, leg muscle strength and handgrip strength in healthy community-dwelling older men (n = 49) and women (n = 48).

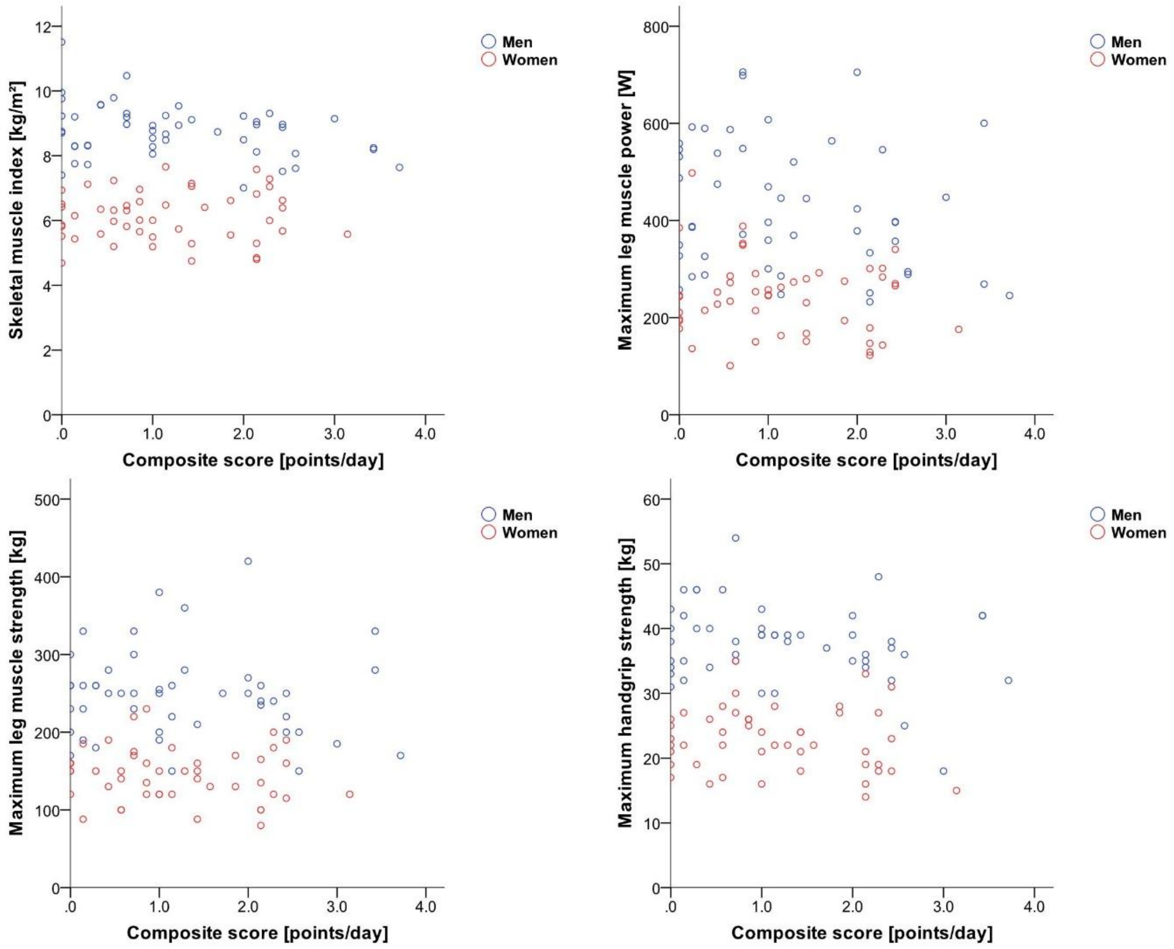

Supplementary figure S5: Scatterplots of association of mean composite score representing protein intake pattern and muscle mass, leg muscle power, leg muscle strength and handgrip strength in healthy community-dwelling older men (n = 49) and women (n = 48).
